# An Evaluation of DNA Methylation Levels and Sleep in Relation to Hot Flashes: A Cross-Sectional Study

**DOI:** 10.3390/jcm13123502

**Published:** 2024-06-15

**Authors:** Ipek Betul Ozcivit Erkan, Hasan Hakan Seyisoglu, Gulcin Benbir Senel, Derya Karadeniz, Filiz Ozdemir, Aysel Kalayci, Mehmet Seven, Neslihan Gokmen Inan

**Affiliations:** 1 Department of Obstetrics and Gynaecology, Cerrahpasa Faculty of Medicine, Istanbul University-Cerrahpasa, Cerrahpaşa Mah. Kocamustafapaşa Cad. No:34/E Fatih/İSTANBUL, Istanbul 34098, Turkey; hasanhakan.seyisoglu@iuc.edu.tr; 2 Sleep Disorders Units, Department of Neurology, Cerrahpasa Faculty of Medicine, Istanbul University-Cerrahpasa, Istanbul 34098, Turkey; gulcin.benbir@iuc.edu.tr (G.B.S.); derya.karadeniz@iuc.edu.tr (D.K.); 3 Department of Medical Genetics, Cerrahpasa Faculty of Medicine, Istanbul University-Cerrahpasa, Istanbul 34098, Turkey; filiz.ozdemir@iuc.edu.tr (F.O.); aysel.kalayci@iuc.edu.tr (A.K.); mimseven@gmail.com (M.S.); 4 Department of Computer Engineering, College of Engineering, Koc University, Istanbul 34450, Turkey; ninan@ku.edu.tr

**Keywords:** hot flashes, epigenetics, DNA methylation, LINE-1 methylation, ALU methylation, sleep

## Abstract

**Objectives:** We aimed to evaluate the DNA methylation levels in perimenopausal and postmenopausal women, measured through Long Interspersed Element-1 (LINE-1) and Alu, and the sleep parameters in relation to the presence of hot flashes (HFs). **Methods:** This cross-sectional study included 30 peri- or postmenopausal women aged between 45 and 55. The menopausal status was determined according to STRAW + 10 criteria and all participants had a low cardiovascular disease (CVD) risk profile determined by Framingham risk score. The sample was divided into two groups based on the presence or absence of HFs documented in their medical history during their initial visit: Group 1 (n = 15) with HFs present and Group 2 (n = 15) with HFs absent. The patients had polysomnography test and HFs were recorded both by sternal skin conductance and self-report overnight. Genomic DNA was extracted from the women’s blood and methylation status was analyzed by fluorescence-based real-time quantitative PCR. The quantified value of DNA methylation of a target gene was normalized by β-actin. The primary outcome was the variation in methylation levels of LINE-1 and Alu and sleep parameters according to the presence of HFs. **Results**: LINE-1 and Alu methylation levels were higher in Group 1 (HFs present), although statistically non-significant. LINE-1 methylation levels were negatively correlated with age. Sleep efficiency was statistically significantly lower for women in Group 1 (HFs present) (74.66% ± 11.16% vs. 82.63% ± 7.31%; *p* = 0.03). The ratio of duration of awakening to total sleep time was statistically significantly higher in Group 1 (HFs present) (22.38% ± 9.99% vs. 15.07% ± 6.93, *p* = 0.03). Objectively recorded hot flashes were significantly higher in Group 1 (4.00 ± 3.21 vs. 1.47 ± 1.46, *p* = 0.03). None of the cases in Group 2 self-reported HF despite objectively recorded HFs during the polysomnography. The rate of hot flash associated with awakening was 41.4% in the whole sample. **Conclusions:** Women with a history of hot flashes exhibited lower sleep efficiency and higher awakening rates. Although a history of experiencing hot flashes was associated with higher LINE-1 and Alu methylation levels, no statistical significance was found. Further studies are needed to clarify this association. This study was funded by the Scientific Research Projects Coordination Unit of Istanbul University-Cerrahpasa. Project number: TTU-2021-35629.

## 1. Introduction

Vasomotor symptoms (VMSs), or hot flashes (HFs), are the most common symptoms observed during menopausal transition, affecting approximately 80% of menopausal women [1]. HFs are triggered by small elevations in the body temperature within a narrowed thermoneutral zone caused by low levels of estrogen during menopause [2]. With a median duration of 7.4 years, VMSs are considered as a subclinical cardiovascular disease (CVD) risk factor [3]. HFs which awaken women during the night disturb the parasympathetic milieu of sleep, making VMSs a significant factor contributing to sleep disturbances among menopausal women [4]. Additionally, VMSs are associated with a higher risk of obstructive sleep apnea (OSA) in midlife women, a condition known to disrupt sleep [5]. OSA is also associated with mood disorders, which have an impact on sleep [6].

Epigenetics, a reversible gene regulation process where changes in gene expression occur without alterations in the deoxyribonucleic acid (DNA) sequence, has emerged as a relevant method to demonstrate the link between disease and its individual-based causes [7]. Previous epigenetic studies have investigated the relationship between VMS, menopausal age, and biological and epigenetic aging [8,9]. Thurston et al. demonstrated that severe or late-occurring HFs were associated with accelerated epigenetic aging among postmenopausal women after adjusting for chronological age, race/ethnicity, hysterectomy, education, body mass index (BMI), smoking and sleep disturbance. [8]. The hypermethylation of receptor activator of nuclear factor kappa beta and follicle-stimulating hormone (FSH) receptor genes has been shown with the presence of HF in postmenopausal women [10]. Accelerated epigenetic aging has been associated with severe and late-occurring VMSs [8]. However, methylation levels observed at repeating elements such as Alu and Long interspersed element-1 (LINE-1), which are whole-genome markers, have not been studied in relation to HF. These two markers, representing 10% of the whole genome, are active mobile elements within the human genome. The typical Alu sequence is approximately 300 nucleotides in length and is distinguished by its high CG and CpG content. LINE-1, belonging to the LINEs family of repetitive sequences, is estimated to have approximately 700,000 copies in the human genome [11]. They play crucial roles in regulating tissue-specific genes, modulating gene expression, controlling cell division and differentiation, and establishing genome architecture [12,13]. Currently, the methylation levels of these repeating elements, which reflect global DNA methylation, are used for the assessment of malignancy and cardiovascular and neurodegenerative disease risk [14]. LINE-1 and Alu elements were shown to have associations with atherosclerotic disease, stroke, and ischemic heart disease [15,16]. Previous studies have documented low methylation levels in global DNA and LINE-1 in individuals at higher cardiovascular disease risk or with ischemic heart disease [17,18].

DNA methylation levels and sleep parameters may vary among peri- and postmenopausal women experiencing hot flashes. Therefore, in this cross-sectional study, we aimed to evaluate the DNA methylation levels in perimenopausal and postmenopausal women, measured through LINE-1 and Alu, and the sleep parameters in relation to the presence of HFs.

## 2. Materials and Methods

This cross-sectional study was conducted at the gynecological endocrinology clinic of Istanbul University-Cerrahpasa, Cerrahpasa Faculty of Medicine between December 2020 and December 2022, following STROBE guidelines. Peri- or postmenopausal women aged between 45 to 55 years were recruited [19]. Participants were required to be non-smokers and fulfill specific hormonal and biochemical criteria: follicle-stimulating hormone levels > 35 IU/L, estradiol levels < 20 pg/mL, high sensitive c-reactive protein levels < 5 mg/dL, fasting blood glucose < 90 mg/dL, fasting insulin levels < 37.06 µIU/mL, body mass index < 30 kg/m^2^, thyroid stimulating hormone < 4.2 mIU/L, free thyroxine < 1.7 ng/dL, blood pressure < 140/90 mmHg measured twice with a 10 min interval, and low CVD risk estimated by the Framingham General Cardiovascular Risk Score (10-year risk) (<10%) [20]. Additionally, vitamin D levels > 20 (µg/L), folic acid levels ranging from 3.1 to 17.5 ng/mL, and vitamin B12 levels between 200 pg/mL and 900 pg/mL were required. The researchers selected hormonal and biochemical criteria based on previous studies to ensure that participants with a low cardiovascular disease (CVD) risk profile were included and to standardize parameters that might influence methylation levels [21,22,23,24,25,26,27,28]. Women meeting any of the following criteria were excluded from the study: a history of CVD (including myocardial infarction, stroke, arrhythmia), hypertension, diabetes, surgically induced menopause, recent hormonal treatment within the last 3 months, obstructive sleep apnea, or the use of medication or pre-existing diagnoses of sleep disorders or conditions known to impact sleep quality, such as depression, that could disrupt sleep.

Following the recruitment of 47 women, a total of 30 participants were included and divided into two groups based on the presence of hot flashes in their medical history documented during their initial visit. According to the STRAW criteria, menopause was defined as the permanent cessation of menses for a complete year, while perimenopause was defined as occurring in women with FSH levels above 25 IU/L and an interval of amenorrhea exceeding 60 days [19]. Later, during a polysomnography (PSG) test, HFs were recorded using sternal skin conductance (objective method) and self-report (subjective method) at the Sleep and Disorders Unit of the Neurology Department. Peripheral venous blood samples were collected from the participants the morning after the PSG test to assess DNA methylation levels in the molecular laboratory of the Medical Genetics Department. Additionally, the Pittsburgh Sleep Quality Index (PSQI), consisting of 24 questions in a validated Turkish form [29], was administered to evaluate sleep quality. The global PSQI score, derived from the sum of seven component scores assessing subjective sleep quality, sleep latency, sleep duration, habitual sleep efficiency, sleep disturbances, use of sleeping medication, and daytime dysfunction, ranges from 0 to 21, with higher scores indicating poorer sleep quality. The Epworth Sleepiness Scale comprises eight items, each assessing the likelihood of falling asleep during daily activities, with responses graded on a scale of 0 to 3. Total scores of 10 or higher suggest hypersomnolence [30].

A detailed explanation was given to all women, and informed consents were obtained in compliance with the Declaration of Helsinki. The institutional ethics committee and reviewer board authorized the presented study (08/12/2020-160382). This study was funded by the Scientific Research Projects Coordination Unit of Istanbul University-Cerrahpasa. Project number: TTU-2021-35629. This study has been registered at clinicaltrials.gov.tr with the NCT number of NCT05892211. The full trial protocol can be accessed through https://clinicaltrials.gov/study/NCT05892211 (accessed on 13 June 2024).

The primary outcome of our study is the variation in the methylation levels of LINE-1 and Alu and the sleep parameters in relation to the presence of hot flashes.

### 2.1. Epigenetic Analysis

Genomic DNA was extracted from whole blood samples using a commercial isolation kit (Macherey-Nagel, Dueren, Germany). All isolation steps were carefully performed according to the manufacturer’s instructions. DNA concentration was measured by using a Nanodrop 2000 spectrophotometer (Thermo Fisher Scientific, Waltham, MA, USA).

DNA treatment with bisulfite leads to the deamination of unmethylated cytosines while leaving methylated cytosines unchanged. Bisulfite modification from the DNA specimens was performed based on the manufacturer’s protocol using a DNA methylation kit (Epitect Fast DNA Bisulfite Kit, Qiagen, Germantown, MD, USA) and eluted into 20 μL Elution buffer. A non-CpG cytosine residue was used as an internal control to confirm bisulfite DNA conversion efficiency (99%). The bisulfite treatment was performed using an Applied Biosystem VeritiThermal Cycler (Applied Biosystems; Thermo Fisher Scientific, Inc., Waltham, MA, USA) with the following thermocycling conditions: initial denaturation step at 95 °C for 5 min; incubation at 60 °C for 25 min, second denaturation step at 95 °C for 5 min, incubation at 60 °C for 85 min; third denaturation step at 95 °C for 5 min; incubation at 60 °C for 175 min; and hold at 20 °C for 10 min. Purified DNA was eluted in buffer AE and stored at −20 °C for use in subsequent experiments.

To analyze Alu and LINE-1 methylation status, we used a fluorescence-based, real-time quantitative polymerase chain reaction (PCR) with the MethyLight method. It can be used to detect methylated DNA sequences in the presence of the unmethylated DNA. In this study, the MethyLight method was carried out using two primers and a TaqMan probe for each gene. The study was carried out with methylated primers for ALU/LINE and with unmethylated primers for β-actin, which is the control gene. The primers and probes were used according to the previously reported references as listed in Table S1 [31,32]. The qPCR reactions were performed in a Real-Time PCR system (Rotor-Gene Q, Qiagen, Hilden, Germany) using the EpiTect^®^ MethyLight PCR (Qiagen, Hilden, Germany) according to the manufacturer’s protocol. The reactions were performed in a volume of 20 μL that included the following: 2X EpiTect MethyLight Master mix 10 μL containing 1 μL 10 µM primer–probe mix with 0.5 µL probe; 1 μL bisulfite-treated DNA template (80 ng); and 6.5 μL RNase-free water. Cycle conditions were as follows: initial PCR activation step at 95 °C for 5 min, 95 °C for 15 s (denaturation step), and 60 °C for 1 min (combined annealing/extension step with fluorescence data collection) for 40 cycles. The quantified value of DNA methylation of a target gene was normalized by β-actin because it is used as a control, as it remains stable in the presence of a wide range of compounds and is expressed within all eukaryotic cell types. Relative copy numbers of Alu and LINE-1 (Alu and LINE-1 delta Ct) were calculated according to the formula [2^−ΔCt^, (ΔCt = CtAlu/LINE-1 − Ctβ-actin)] [9].

### 2.2. Polysomnography and Measurement of Sternal Skin Conductance

PSG was conducted through 3-channel electroencephalography (F4-M1, C4-M1, O2-M1), 2-channel electrooculography, surface chin electromyography (EMG), surface right and left anterior tibial EMG, body position, oronasal thermal sensor, nasal pressure transducer, thoracoabdominal respiratory inductance plethysmography belts for determining the respiratory effort, electrocardiography, pulse, record of respiratory sounds, oxygen saturation, and synchronous video recording. Awakening and sleep stage scoring were calculated, and sleep-related events were defined according to the criteria of the American Academy of Sleep Medicine. The disorders of sleep were diagnosed according to the International Classification of Sleep Disorders [33].

Sympathetic skin response recording was performed with PSG simultaneously throughout the whole night with 4-channel EMG. Sympathetic skin response was recorded at the following site: sternum (active and reference electrode 2 cm apart) [34]. The maximum amplitude and mean latency of HF were calculated (objectively recorded HF, objective method). Sympathetic skin response duration, the corresponding sleep stages of HF episodes, and the association of HF with awakening were noted. Additionally, participants were asked to press a button at their side and self-report when they were woken up by an HF episode during the night (subjectively documented HF, subjective method). The PSG findings of the participants were interpreted by a sleep specialist who was blind to the study group.

### 2.3. Statistical Analysis

The statistical analysis was performed using Statistical Package for Social Sciences version Mac 21. Since there was not a similar study conducted previously, the sample size for this pilot study was accepted as adequate according to the literature [35]. The demographic data were demonstrated with descriptive statistics. Categorical variables were presented as numbers and percentages. The continuous variables were evaluated in terms of the normality of distribution by a Kolmogorov–Smirnov test. Continuous variables were presented as mean ± standard deviation and median (min–max or interquartile range). Statistical comparisons were conducted using a Mann–Whitney U for non-normally distributed data and an independent sample *t*-test for normally distributed data. Scores, numbers, ratings, and variables with wide standard deviations were compared with the Mann–Whitney U test even when normally distributed. The relationship between the two variables was calculated with Pearson correlation analysis. The rate of HF episodes in any sleep stage was initially calculated without adjusting for the time spent in each stage. Following that, we adjusted the number of HF episodes for the duration of time spent in each stage. This adjustment involved calculating the unit percent HF episode rate for each case. Statistical significance was accepted as *p <* 0.05.

## 3. Results

**Figure 1:**
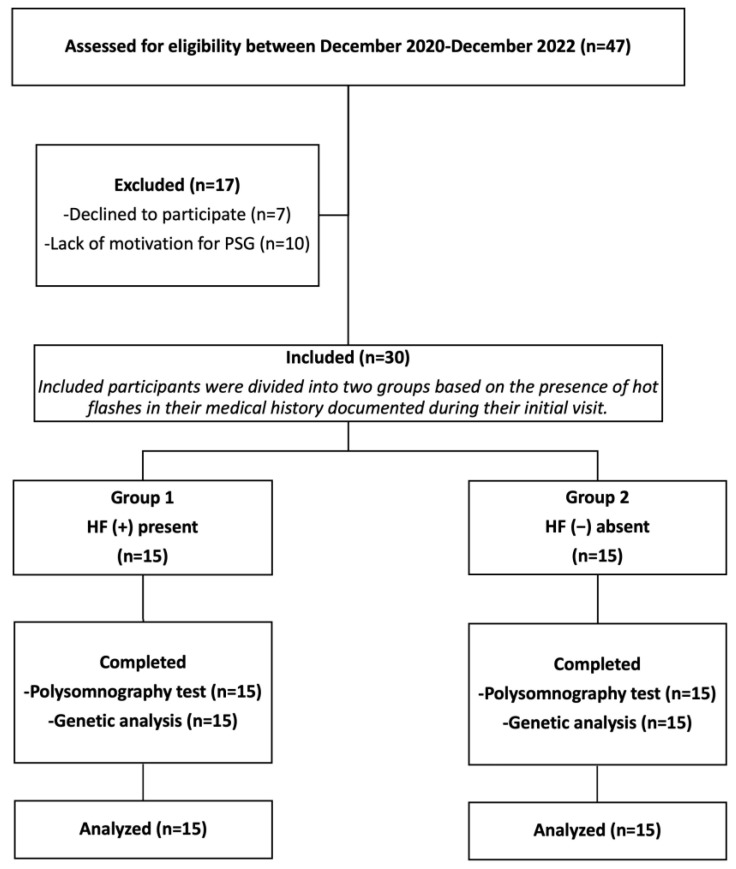
The flow diagram of the study.

Figure 1 illustrates the recruitment process. Initially, a total of 47 women were recruited, but 17 of them declined to participate, mainly due to the requirement of sternal skin conductance and PSG recording, which necessitated an overnight hospital stay. The patients were divided into two groups based on the presence of hot flashes in their medical history obtained during their initial visit, before conducting any tests. Table 1 presents the demographic and clinical characteristics of the patients. The mean age of women in both groups was comparable (50.57 ± 3.46 vs. 50.69 ± 2.66; *p* = 0.81, respectively). Group 1 had only 4 perimenopausal women, while Group 2 had only 3 perimenopausal women (*p* = 0.50). The mean time since the onset of menopause was also similar between the two groups (3.00 ± 2.77 vs. 3.46 ± 2.50; *p* = 0.68, respectively). None of the women experienced surgically induced menopause.

The quantification of methylation in samples is presented in Figure S1, and the LINE-1 and Alu methylation levels are described by delta Ct in Table 2. The mean values of LINE-1 Delta Ct were 1.03 ± 0.40 vs. 0.73 ± 0.29 for Group 1 and Group 2, respectively (*p* = 0.06). The mean values of Alu Delta Ct were comparable between both groups (272.68 ± 457.20 vs. 77.44 ± 95.69 (*p* = 0.38). For the whole sample, the 95% confidence intervals for LINE-1 and Alu methylation levels were 0.74–1.02 and 48.33–301.80, respectively.

The results indicated that DNA methylation levels of both Alu and LINE-1 were higher in the HF (+) group than in the HF (−) group. However, these differences were not statistically significant. For the whole cohort, LINE-1 methylation levels exhibited a low negative correlation with age (r = −0.40, *p* = 0.03), although there was no correlation between LINE-1 and Alu DNA methylation levels and total HF episode number during the night, rate of subjective complaints accompanying objectively documented HFs, duration and amplitude of HF episodes, and duration of awakening. There was no statistically significant difference between mean LINE-1 and Alu Delta Ct levels in the HF (+) group according to the self-reported HFs accompanying objectively recorded HFs during the PSG.

The PSG findings are presented in Table 3. The total sleeping duration was shorter in Group 1, but the difference was not statistically significant (355.57 ± 54.40 min vs. 387.57 ± 43.23 min, respectively; *p* = 0.09). The sleep efficiency was statistically significantly lower in Group 1 compared to Group 2 (74.66% ± 11.16% vs. 82.63% ± 7.31%, respectively; *p* = 0.03). The distribution of sleep stages was comparable between the two groups. However, the ratio of duration of awakening to total sleep time was statistically significantly higher in Group 1 (22.38% ± 9.99% vs. 15.07% ± 6.93; *p* = 0.03). The PSQI score was higher in Group 1, which showed poorer sleep quality (9.33 ± 3.15 vs. 6.40 ± 3.78, respectively; *p* = 0.04) (Table 3). No other sleep disorders were diagnosed in our cohort based on PSG findings.

The parameters related to recorded HFs during the PSG test are presented in Table 4. For the entire cohort, the mean rate of self-reported HFs accompanying objectively recorded HFs is 9.13 ± 19.50%. None of the cases in Group 2 self-reported HFs, despite objectively recorded HFs during the PSG (*p* = 0.003). Objectively recorded HFs were present in both groups although statistically higher in Group 1 compared to Group 2 (4.00 ± 3.21 vs. 1.47 ± 1.46, respectively, *p* = 0.03). The rate of HFs associated with awakening was 41.4% in the whole sample. HFs were mostly observed at Non-Rapid Eye Movement 2 (NREM2) and NREM3 stages during the recording session (47.56% vs. 31.71%) followed by wake (9.76%), NREM1 (9.76%), and REM (1.22%). When the rate of HFs was calculated considering the duration of sleep stages, NREM3 (46.48%) was found to be the most common sleep stage where HF occurred. The maximum amplitude value, longest duration, and mean of the total duration of HF episodes, as well as the maximum, mean, and total duration of wake, were significantly higher for women in Group 1 (Table 4). The self-reported HFs accompanying objectively documented HFs were positively correlated with mean and maximum HF amplitude, mean and maximum duration of HF, and mean and maximum duration of wake (r = 0.60, *p* = 0.001; r = 0.65, *p <* 0.001; r = 0.58, *p* = 0.001; r = 0.66, *p <* 0.001; r = 0.55, *p* = 0.002; r = 0.60, *p <* 0.001) (Table 5).

## 4. Discussion

In this study, we investigated the alterations in DNA methylation levels of LINE-1 and Alu and sleep patterns in perimenopausal and postmenopausal women with respect to HFs. We demonstrated that HFs disrupted sleep even in the absence of accompanying sleep disorders. Interestingly, women who did not report experiencing HFs in their medical history during recruitment were found to have objective HF episodes recorded during the PSG test. However, none of these women self-reported these HF episodes, despite their objective documentation during the PSG test. This finding highlights the importance of objective measures in accurately assessing the prevalence of HFs. Additionally, we observed for the first time in the literature that the methylation levels of Alu and LINE-1 were higher in peri- and postmenopausal women with a history HFs, although statistically non-significant. Furthermore, the methylation levels were negatively correlated with age.

Recent studies have identified an association between CVD development and poor sleep patterns [6,36,37]. Specifically, repetitive arousals were shown to disrupt sympathovagal modulation, leading to increased blood pressure and contributing to adverse cardiovascular outcomes such as endothelial dysfunction and increased arterial stiffness [38]. Baker et al.’s study also reported disrupted autonomic nervous system balance, demonstrating that arousals caused by nocturnal hot flashes increased heart rate and blood pressure [39]. Consequently, disrupted cardiovascular restoration during the deepest stages of sleep of NREM due to HF-induced arousals may increase sympathetic activity overnight, resulting in decreased cardiac autonomic vagal activity and ultimately poor cardiovascular outcomes [40]. In our cross-sectional study, we found that HFs were mainly observed during NREM2 and NREM3 stages and were associated with unfavorable objective and subjective sleep outcomes. However, the potential impact of hot flashes on CVD risk, mediated by disruptions in both objective and subjective sleep patterns and prolonged periods of nocturnal wakefulness, warrants further investigation through longitudinal studies.

Our study facilitated the comparison of objectively recorded HFs with self-reported ones by objectively recording HFs through sternal skin conductance during PSG. It is well-established that physiologically measured HFs often do not correlate with the HFs reported retrospectively the following morning [41]. Our findings revealed that HF episodes were objectively documented even in women who did not report having HFs in their medical history, thus highlighting the discrepancy between anamnesis-based and objectively recorded HF episodes. Further investigation is warranted to explore the potential association between HF occurring without the patient’s conscious awareness and adverse cardiovascular outcomes. While both self-reporting and sternal skin conductance are recognized as valid measures of VMS, the search for a superior screening method for identifying VMS severity and VMS-associated CVD risk continues. Artificial intelligence could play a crucial role in identifying severe VMS, as demonstrated by Maniaci et al. for patients with symptoms related to obstructive sleep apnea [6]. They demonstrated that artificial intelligence could be advantageous in assessing patients with OSA-related symptoms and determining the risk of disease severity using clinic-based algorithms. By utilizing long-term follow-up and demographic data, a prediction model for VMS severity can be developed through machine learning.

There may be an association between HFs and DNA methylation levels which could help to understand the potential link between HFs with CVD. HFs are recognized as a subclinical risk factor of CVD, as evidenced by their association with low flow-mediated dilation, coronary artery and aortic calcifications, and increased carotid intima-media thickness [3,42]. LINE-1 and Alu elements were selected as markers for methylation analysis due to their reported associations with atherosclerotic disease, stroke, and ischemic heart disease [15,16]. Previous studies have documented low methylation levels in global DNA and LINE-1 in individuals at higher cardiovascular disease risk or with ischemic heart disease [17,18]. Our findings, for the first time, demonstrated higher LINE-1 and Alu methylation levels in women with a history of HFs. However, this association was not statistically significant. This may be attributed to the relatively young age of our study population [9,16,17], the cross-sectional design, and the absence of a CVD-present group in our sample. Moreover, the presence of HFs itself, although considered a subclinical CVD risk factor, may not impact methylation, or methylation processes may not impact one’s susceptibility/experience of HFs. Additionally, factors such as genomic instability, individual differences, and various diseases may have contributed to the discrepancies in our results [17,43,44]. Nonetheless, we also confirmed that methylation levels decrease with age [45]. Therefore, conducting longitudinal and large-scale epidemiological studies among women with and without cardiovascular events may yield more reliable results to understand the relationship between HFs and DNA methylation levels. Moreover, the association between sleep deprivation and changes in the epigenome has been investigated [46]. An association has been identified between increased estrogen receptor methylation and sleep problems, suggesting that ESR1 methylation may be linked to vasomotor symptoms and consequently contribute to increased sleep problems in healthy middle-aged and older women [47]. This association could be further analyzed in further studies, in addition to its potential connection to CVD.

To the best of our knowledge, this is the first study to analyze whole genome markers, LINE-1 and Alu, and their methylation levels in women with HFs. It is also unique in its use of both objective (PSG and sternal skin conductance) and subjective (self-reported HF at night) methods to demonstrate the impact of hot flashes on sleep. Additionally, we aimed to show the pure association of HFs, methylation levels, and sleep, which is why we homogenized our groups in terms of age and estimated CVD risk using the Framingham General Cardiovascular Risk Score [20]. The main limitations of our study were the limited sample size, cross-sectional design, and lack of long follow-up. The findings of this study, especially the borderline significant findings, should be confirmed by a subsequent study. The subsequent studies should determine their sample sizes according to the results of this study and obtain high power. Another concern was the high confidence intervals seen in LINE-1 and Alu methylation levels. Therefore, future studies should focus on studying global DNA instead of nuclear elements. DNA methylation may also have been affected by confounding factors such as lifestyle, diet, alcohol consumption, and body mass index, which could account for bias.

## 5. Conclusions

Hot flashes have an impact on sleep parameters in perimenopausal and postmenopausal women. These sleep disturbances were evident in our sample, which had low CVD risk and no accompanying sleep disorders. Women with a history of hot flashes exhibited lower sleep efficiency and higher awakening rates. Although a history of experiencing hot flashes was associated with higher LINE-1 and Alu methylation levels, no statistical significance was found. Further studies with longer follow-up time are needed with larger and more diverse populations to clarify this association. Additionally, investigating potential confounding factors such as lifestyle, diet, and BMI may help elucidate the underlying mechanisms of HFs, epigenetic changes, and cardiovascular health.

## Figures and Tables

**Table 1:** Demographic and clinical characteristics of the participants.

Variables	Group 1 HF (+) (n = 15)	Group 2 HF (−) (n = 15)	*p*
Age	50.57 ± 3.46	50.69 ± 2.66	0.81 ^a^
BMI (kg/m^2^)	25.15 ± 3.56	24.32 ± 2.25	0.68 ^a^
Time since onset of menopause (yrs)	3.00 ± 2.77	3.46 ± 2.85	0.71 ^b^
FSH (IU/L)	94.21 ± 29.02	82.26 ± 16.68	0.14 ^a^
E_2_ (pmol/L)	10.51 ± 6.45	10.03 ± 5.19	0.81 ^b^
Framingham score (%)	3.67 ± 1.82	3.94 ± 1.80	0.95 ^b^
SBP (mmHg)	115.71 ± 14.20	113.85 ± 15.30	0.72 ^a^
DBP (mmHg)	75.64 ± 8.82	73.15 ± 11.42	0.32 ^b^
MBP (mmHg)	89.00 ± 10.27	86.77 ± 12.22	0.45 ^a^
Glucose (mg/dL)	84.50 ± 7.50	88.23 ± 8.76	0.15 ^a^
Insulin (µIU/mL)	7.36 ± 1.69	7.62 ± 2.29	0.54 ^a^
HbA1C (%)	5.71 ± 0.27	5.70 ± 0.23	0.76 ^a^
HDL (mg/dL)	64.64 ± 15.23	73.15 ± 13.09	0.37 ^a^
LDL (mg/dL)	133.29 ± 33.72	147.31 ± 29.40	0.31 ^a^
Total cholesterol (mg/dL)	202.36 ± 39.31	229.69 ± 25.03	0.13 ^a^
Triglyceride (mg/dL)	98.64 ± 48.21	91.77 ± 48.40	0.24 ^b^
TSH (mIU/L)	2.06 ± 1.10	1.79 ± 0.93	0.31 ^a^
T_4_ (ng/dL)	1.23 ± 0.16	1.27 ± 0.21	0.21 ^a^
CRP (mg/L)	0.94 ± 0.65	1.53 ± 1.64	0.26 ^b^
Folic acid (ng/mL)	9.56 ± 3.64	9.89 ± 3.47	0.63 ^b^
Vitamin B12 (pg/mL)	454.85 ± 236.10	505.71 ± 289.80	0.81 ^b^
Vitamin D (µg/L)	23.74 ± 6.66	26.97 ± 15.67	0.87 ^b^

BMI: body mass index, CRP: C-reactive protein, DBP: diastolic blood pressure, E_2_: estradiol, FSH: follicle-stimulating hormone, HDL: high-density lipoprotein, LDL: low-density lipoprotein, MBP: mean blood pressure, SBP: systolic blood pressure, T_4_: thyroxine; ^a^ Calculated by independent sample *t*-test; ^b^ Calculated by Mann–Whitney U test.

**Table 2:** ALU and LINE-1 methylation levels.

Variables	Group 1 HF (+) (n = 15)		Group 2 HF (−) (n = 15)		*p*
	Mean ± SD	Median (IQR)	Mean ± SD	Median (IQR)	
ALU Delta Ct	272.68 ± 457.20	39.40 (308.53)	77.44 ± 95.69	17.75 (148.68)	0.38 ^a^
LINE-1 Delta Ct	1.03 ± 0.40	0.93 (0.54)	0.73 ± 0.29	0.77 (0.36)	0.06 ^a^

^a^ Mann–Whitney U test was used.

**Table 3:** The polysomnography findings and subjective sleep quality index results of women.

Variables	Group 1 HF (+) (n = 15)	Group 2 HF (−) (n = 15)	*p*
Total recording time (min)	476.14 ± 20.85	468.49 ± 22.25	0.34 ^a^
Total sleep time (min)	355.57 ± 54.40	387.57 ± 43.23	0.09 ^a^
Sleep latency (min)	19.01 ± 23.09	12.91 ± 7.91	0.69 ^b^
Sleep efficiency (%)	74.66 ± 11.16	82.63 ± 7.31	**0.03** ^a^
	95% CI: 68.68–80.84	95% CI: 78.58–86.68	
REM sleep latency (min)	137.47 ± 88.72	123.70 ± 63.13	0.63 ^a^
Apnea hypopnea index (/hr)	6.60 ± 5.81	7.41 ± 7.18	0.77 ^b^
PLMS	2.25 ± 4.31	6.91 ± 11.07	0.16 ^b^
NREM1 (%)	9.68 ± 4.05	7.89 ± 1.96	0.14 ^a^
NREM2 (%)	38.39 ± 9.38	43.98 ± 7.41	0.08 ^a^
NREM3 (%)	14.67 ± 5.93	15.69 ± 4.69	0.61 ^a^
REM (%)	14.88 ± 5.34	17.39 ± 4.58	0.18 ^a^
Wake (%)	22.38 ± 9.99	15.07 ± 6.93	**0.03** ^a^
	95% CI: 16.85–27.91	95% CI: 11.23–18.90	
Minimum O_2_ saturation (%)	88.27 ± 4.25	89.40 ± 3.29	0.42 ^a^
Mean O_2_ saturation (%)	95.77 ± 1.23	95.77 ± 1.12	0.99 ^a^
Maximum heart rate (bpm)	121.76 ± 21.19	120.78 ± 18.45	1.00 ^a^
Mean heart rate (bpm)	60.71 ± 7.53	64.66 ± 17.58	0.43 ^a^
Epworth sleepiness scale	4.13 ± 2.36	3.73 ± 3.69	0.38 ^b^
PSQI	9.33 ± 3.15	6.40 ± 3.78	**0.04** ^b^
	95% CI: 7.59–11.08	95% CI: 7.59–11.08	

bpm: beat per minute, CI: confidence interval, NREM: non-rapid eye movement stage, PLMS: periodic leg movements during sleep, PSQI: Pittsburgh Sleep Quality Index, REM: rapid eye movement stage. ^a^ Independent sample *t*-test was used. ^b^ Mann–Whitney U test was used. Bold values denote statistical significance at the *p* < 0.05 level.

**Table 4:** The parameters regarding objectively recorded hot flashes of the participants.

Variables	Group 1 HF (+) (n = 15)	Group 2 HF (−) (n = 15)	*p*
Total number of HF episodes	4.00 ± 3.21	1.47 ± 1.46	**0.03** ^b^
	95% CI: 2.22–5.78	95% CI: 0.66–2.27	
Number of HF episodes during sleep	3.60 ± 3.00	1.33 ± 1.50	**0.02** ^b^
	95% CI: 1.94–5.26	95% CI: 0.50–2.16	
Number of HF episodes during wake	0.40 ± 1.06	0.13 ± 0.52	0.31 ^b^
Rate of self-reported HFs accompanying objectively recorded HF (%)	18.27 ± 24.68	0	**0.003** ^b^
	95% CI: 4.60–31.93		
Maximum amplitude of HF (µmho)	11.39 ± 7.01	6.27 ± 5.74	**0.04** ^a^
	95% CI: 7.51–15.27	95% CI: 3.09–9.45	
Mean amplitude of HF (µmho)	9.29 ± 5.82	5.69 ± 5.15	0.08 ^a^
Maximum duration of HF (s)	33.74 ± 24.59	11.09 ± 10.45	**0.003** ^b^
	95% CI: 20.12–47.35	95% CI: 5.31–16.88	
Mean duration of HF (s)	51.80 ± 116.05	9.96 ± 9.47	**0.007** ^b^
Rate of HF associated with awakening (%)	44.60 ± 43.48	27.20 ± 40.63	0.21 ^b^
Maximum duration of wake (min)	4.00 ± 4.35	0.53 ± 1.16	**0.02** ^b^
	95% CI: 1.59–6.41	95% CI: −0.11–1.17	
Mean duration of wake (min)	2.79 ± 3.34	0.46 ± 0.89	**0.02** ^b^
	95% CI: 0.94–4.65	95% CI: −0.3–0.95	
Total duration of wake (min)	8.36 ± 11.89	1.13 ± 3.44	**0.02** ^b^
	95% CI: 1.77–14.94	95% CI: −0.77–3.04	

CI: confidence interval; HF: hot flash. ^a^ Independent sample *t*-test was used. ^b^ Mann–Whitney U test was used. Bold values denote statistical significance at the *p* < 0.05 level.

**Table 5:** The correlation analysis results.

Variables	r	*p*
LINE-1 Delta Ct		
Age	−0.40	**0.03**
Rate of self-reported HFs accompanying objectively recorded HF		
Mean amplitude of HF	0.60	**0.001**
Maximum amplitude of HF	0.65	**<0.001**
Mean duration of wake	0.55	**0.002**
Maximum duration of wake	0.60	**<0.001**

HF: hot flash. Bold values denote statistical significance at the *p* < 0.05 level.

## Data Availability

The data that support the findings of this study are available from the corresponding author, IBOE, upon reasonable request.

## Supplementary Materials

The following supporting information can be downloaded at: https://www.mdpi.com/article/10.3390/jcm13123502/s1, Table S1: Primer and probe sequences for MethyLight PCR [31,32]; Figure S1: The quantification of methylation in samples by using the threshold cycle values (CT) determined by qPCR.
