# Supplementary material for: An Evaluation of DNA Methylation Levels and Sleep in Relation to Hot Flashes: A Cross-Sectional Study
Source: J Clin Med. 2024 Jun 15;13(12):3502. doi: 10.3390/jcm13123502 (PMC11204679; doi:10.3390/jcm13123502)
Supplement: Supplementary file 1 [file jcm-13-03502-s001.zip › Table S1.pdf]

Supplementary Table S1. Primer and probe sequences for MethyLight PCR

Gene		Primer sequence	Ref.
**Alu**	Forward<br>Reverse<br>Probe	5′-GCGCGGTGGTTTACGTTT-3′<br>5′-AACCGAACTAATCTCGAACTCCTAAC-3′<br>5′-6FAM-AAATAATCCGCCCGCCTCGACCT-BHQ1-3′	[31]
**LINE-1**	Forward<br>Reverse<br>Probe	5′-GGACGTATTTGGAAAATCGGG-3′<br>5′-AATCTCGCGATACGCCGTT-3′<br>5′-6FAM-TCGAATATTGCGTTTTCGGATCGGTTT-BHQ1-3′	[31]
**b-actin**	Forward<br>Reverse<br>Probe	5′-TGGTGATGGAGGAGGTTTAGTAAGT-3′<br>5′-AACCAATAAAACCTACTCCTCCCTTAA -3′<br>5′-6FAM-ACCACCACCCAACACACAATAACAAACACA-BHQ1 -3′	[32]
