# Supplementary figures and images for: An Evaluation of DNA Methylation Levels and Sleep in Relation to Hot Flashes: A Cross-Sectional Study
Source: J Clin Med. 2024 Jun 15;13(12):3502. doi: 10.3390/jcm13123502 (PMC11204679; doi:10.3390/jcm13123502)

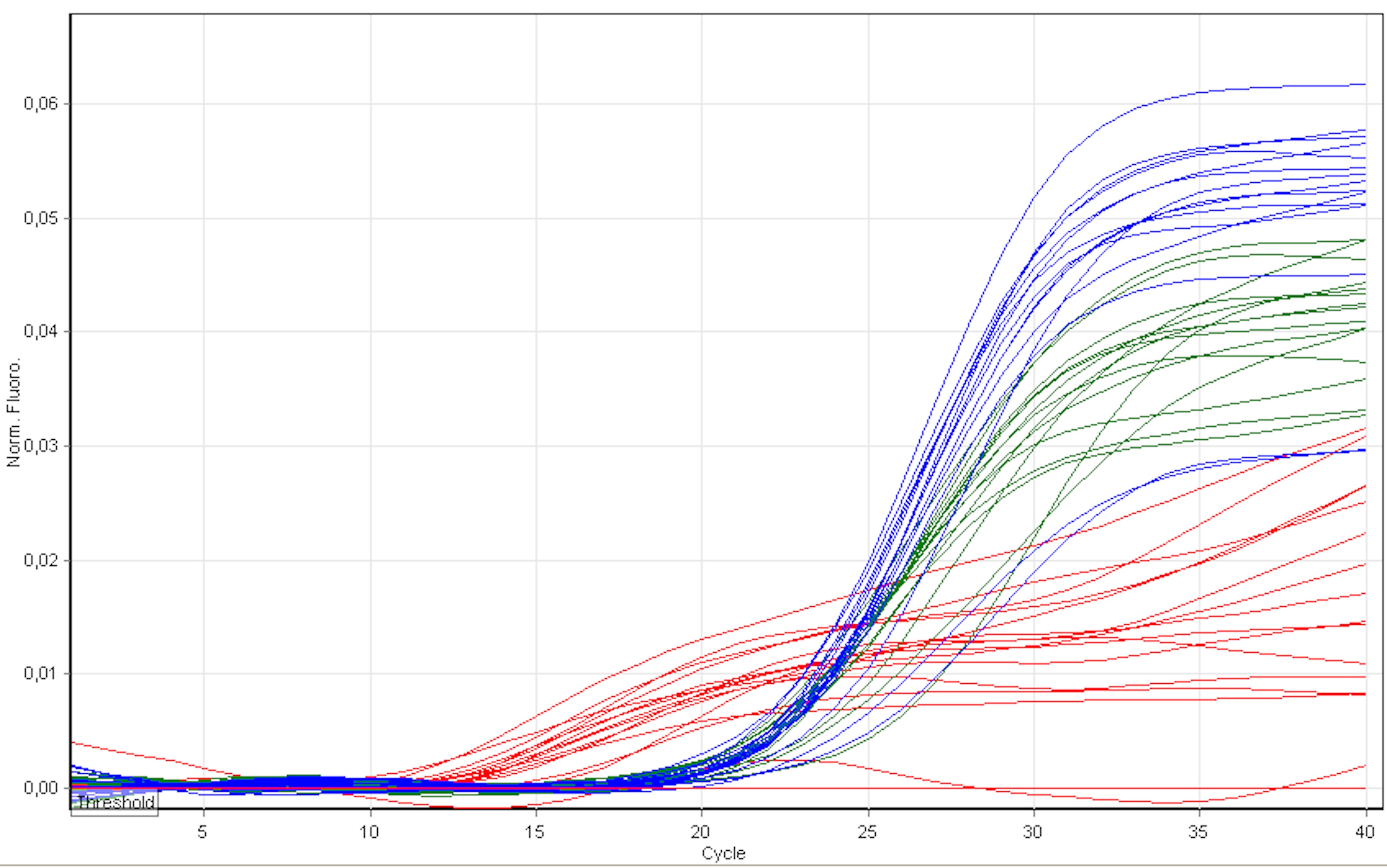

Supplement: Supplementary file 1 [file jcm-13-03502-s001.zip › Figure S1.jpg]
